# Outcomes of carbapenem-resistant *Acinetobacter baumannii* bloodstream infections in intensive care units and prognostic effect of different antimicrobial regimens

**DOI:** 10.1186/s13613-025-01580-7

**Published:** 2025-10-18

**Authors:** Chieh-Lung Chen, Kuang-Yao Yang, Chung-Kan Peng, Ming-Cheng Chan, Chau-Chyun Sheu, Jia-Yih Feng, Sheng-Huei Wang, Wei-Hsuan Huang, Chia-Min Chen, How-Yang Tseng, Yu-Chao Lin, the T-CARE (Taiwan Critical Care andInfection) Group

**Affiliations:** 1 Division of Pulmonary and Critical Care Medicine, Department of Internal Medicine, China Medical University Hospital, China Medical University, No. 2, Yude Road, North District, Taichung, 40402 Taiwan; 2 Department of Public Health, College of Public Health, China Medical University, Taichung, Taiwan; 3 Department of Chest Medicine, Taipei Veterans General Hospital, Taipei, Taiwan; 4 Institute of Emergency and Critical Care Medicine, School of Medicine, National Yang Ming Chiao Tung University, Taipei, Taiwan; 5 Cancer and Immunology Research Center, National Yang Ming Chiao Tung University, Taipei, Taiwan; 6 Division of Pulmonary and Critical Care Medicine, Department of Internal Medicine, Tri-Service General Hospital, National Defense MedicalUniversity, Taipei, Taiwan; 7 Department of Critical Care Medicine, Taichung Veterans General Hospital, Taichung, Taiwan; 8 Department of Post-Baccalaureate Medicine, College of Medicine, National Chung Hsing University, Taichung, Taiwan; 9 Division of Pulmonary and Critical Care Medicine, Department of Internal Medicine, Kaohsiung Medical University Hospital, Kaohsiung Medical University, Kaohsiung, Taiwan; 10 Department of Internal Medicine, School of Medicine, College of Medicine, Kaohsiung Medical University, Kaohsiung, Taiwan; 11 Institute of Emergency and Critical Care Medicine, School of Medicine, National Yang Ming Chiao Tung University, Taipei, Taiwan; 12 Institute of Medical Sciences, College of Medicine, National Defense Medical University, Taipei, Taiwan; 13 Division of Infectious Diseases, Department of Internal Medicine, Taichung Veterans General Hospital, Taichung, Taiwan; 14 School of Medicine, China Medical University, Taichung, Taiwan

**Keywords:** *Acinetobacter baumannii*, Bloodstream infection, Carbapenem-resistant, Intensive care unit, Prognosis

## Abstract

**Background:**

Carbapenem-resistant *Acinetobacter baumannii* (CRAB) poses a significant global threat due to limited therapeutic options and high rates of associated mortality. CRAB-related bloodstream infections (BSIs) in intensive care units (ICUs) represent a major clinical challenge. This study aimed to investigate the clinical outcomes of CRAB-BSIs in ICU settings and evaluate the prognostic effect of different antimicrobial regimens.

**Methods:**

This multicenter, retrospective observational study was conducted at five medical centers in Taiwan and included 393 critically ill patients with CRAB-BSIs between January 2015 and December 2019. Clinical and microbiological outcomes were analyzed. Multivariable regression analysis was used to identify independent prognostic factors for day-28 mortality.

**Results:**

The most common causes of CRAB-BSIs were pneumonia (42.5%) and catheter-related infections (38.7%). The day-28 mortality rate following BSI onset was 56.5%. A higher sequential organ failure assessment (SOFA) score independently predicted increased day-28 mortality. Colistin-based therapy was associated with improved survival outcomes in the original (adjusted hazard ratio [aHR], 0.56; 95% confidence intervals (CI), 0.35–0.88) and time-window bias-adjusted (aHR, 0.59; 95% CI, 0.37–0.94) cohorts. Among patients with pneumonia-related CRAB-BSIs, colistin-based therapy did not significantly improve day-28 survival, whereas sulbactam-based therapy showed survival benefit (aHR, 0.37; 95% CI, 0.15–0.91). Neither carbapenem-based nor tigecycline-based therapies demonstrated a mortality benefit on day 28.

**Conclusion:**

CRAB-BSIs are associated with high mortality in critically ill patients. In settings where novel antibiotics are not available, colistin-based therapy was associated with improved clinical outcomes. Among patients with pneumonia-related CRAB-BSIs, sulbactam-based therapy was associated with lower mortality.

**Supplementary Information:**

The online version contains supplementary material available at 10.1186/s13613-025-01580-7.

## Background

Carbapenem-resistant *Acinetobacter baumannii* (CRAB) has emerged as a significant global health threat, affecting both medical costs and mortality [1, 2]. The increasing prevalence of CRAB, which exceeds 60% in various regions [3–6], presents considerable challenges in clinical management. Bloodstream infections (BSIs) due to CRAB (CRAB-BSIs) are particularly critical healthcare-associated infections, characterized by high morbidity and mortality rates [7, 8], with mortality rates ranging from 40% to 68.5% [9–14]; however, most available data were derived from analyses of small cohorts.

Treatment options for CRAB-BSIs are notably limited owing to the pathogen’s multiple antimicrobial resistance mechanisms, which contribute to poor clinical outcomes [15–18]. Novel antibiotics, such as sulbactam-durlobactam and cefiderocol, have demonstrated considerable therapeutic potential against CRAB infections [19–21]. However, the optimal treatment dosage and duration are still under investigation, and conflicting data regarding their efficacy exist [22, 23]. In addition, access to these novel antibiotics is restricted, posing a significant challenge to the global management of CRAB infections.

Considering these limitations, conventional antimicrobial regimens still play a role in managing CRAB infections [24, 25]. Among these, colistin and sulbactam are the most extensively studied antibiotics [26–29] and are recommended by international guidelines as part of the combination therapy for CRAB infections [18, 30, 31]. However, the literature on CRAB-BSIs in intensive care unit (ICU) populations remains relatively scarce [32–34].

This study aimed to assess the clinical outcomes of critically ill patients with CRAB-BSIs admitted to the ICU and to investigate the prognostic effect of different antimicrobial regimens.

## Methods

### Study design and patient population

This multicenter, retrospective observational study was conducted across five tertiary referral hospitals in Taiwan between January 2015 and December 2019. All consecutive adult patients (aged ≥ 20 years) admitted to the ICUs with a CRAB-positive blood culture were included. Patients who did not receive any antimicrobial therapy or those with insufficient data (incomplete/incorrect date records or missing outcome data) for analysis were excluded (Fig. S1). All patients were followed up until death or hospital discharge. The study protocol adhered to the ethical standards outlined in the Declaration of Helsinki and was reported according to the Strengthening the Reporting of Observational Studies in Epidemiology guidelines. It was approved by the institutional review boards (IRBs) of all participating hospitals (IRB numbers: CMUH110-REC1-139, CE18100A, A202005146_TSGHIRB, 2020-11-006AC, and KMUHIRB-E(I)-20180141). Given the retrospective design and noncollection of personally identifiable information, the respective IRBs waived the requirement for informed consent.

### Definitions and data collection

Bloodstream infections (BSIs) are defined as the presence of positive blood cultures accompanied by systemic clinical signs of infection [4]. BSI sources were identified based on clinical evaluations and microbiological findings as previously reported [35]. Briefly, the sources of BSI were categorized as pneumonia, urinary tract infection, soft tissue infection, or intra-abdominal infection. For patients with central venous catheters (CVCs) who did not have another identifiable source of infection, the BSI was deemed catheter-related. Primary bacteremia was defined as a BSI without an identifiable source, no concurrent infections culturing the same organism(s), and absence of a CVC. Further detailed definitions are provided in the Supplementary Materials. Carbapenem resistance was defined as resistance to one or more carbapenems, including meropenem, imipenem, or ertapenem, in accordance with the guidelines of the Clinical and Laboratory Standards Institute.

Patient demographic characteristics, BSI source, disease severity, antimicrobial therapy, and clinical outcomes were retrospectively collected from electronic medical records. Disease severity was evaluated using the acute physiologic assessment and chronic health evaluation (APACHE) II score on the day of ICU admission and the sequential organ failure assessment (SOFA) score on the day of BSI onset.

### Antimicrobial regimens

Intravenous antibiotics administered for at least 2 days within the first 7 days following CRAB-BSI onset were recorded. To reduce the time-window bias associated with delayed antibiotic initiation, only antibiotics commenced within 3 days of BSI onset were included in the outcome analysis [35]. Combination therapy was defined as the concurrent administration of at least two intravenous antibiotics, overlapping for a minimum of 2 days [35]. The loading dose of intravenous colistin therapy followed definitions from relevant previous studies [36]. During the study period, novel antibiotics such as sulbactam-durlobactam and cefiderocol were unavailable in Taiwan.

### Outcome evaluation

Outcomes of interest included clinical and microbiological responses on days 7, 14, and 28 after BSI onset, and all-cause 28-day mortality. Clinical response to CRAB-BSI treatment was classified as cure (discontinuation of antibiotics for CRAB-BSI with no reinitiation within the following 3 days), improvement (partial remission of symptoms and signs, with either continuation of antibiotics or reinitiation within 3 days after discontinuation), or failure (persistent symptoms, escalation of antimicrobial therapy, or death). Microbiological response was defined as eradication (no pathogen detected in follow-up blood cultures), failure (persistence of the pathogen in subsequent blood cultures), or undetermined (lack of blood cultures during follow-up).

Survival analyses were performed in the entire study cohort and, separately, in a time-window bias-adjusted cohort excluding patients who died on the day of BSI onset.

### Statistical analysis

All statistical analyses were conducted using the IBM SPSS for Windows version 25 (IBM Corp., Armonk, NY, USA) and R statistical software version 4.3.3. Continuous variables were presented as means and standard deviations (SDs) and compared using Student’s *t*-test. Ordinal data were presented as medians and interquartile ranges (IQRs) and compared between groups using the Mann–Whitney *U* test. Categorical variables were described as counts and percentages, and between group differences were assessed using the chi-squared test. Day-28 mortality was evaluated using Kaplan–Meier survival curves and compared with the log-rank test. Cox regression models were used to identify prognostic factors associated with day-28 mortality, and logistic regression was applied to evaluate factors that influence other treatment outcomes. The multivariable regression model included variables with a *p*-value < 0.1 in the univariate analysis and clinically relevant factors, such as patient age and antimicrobial regimens. Results were expressed as hazard ratios (HR) for Cox regression and odds ratios (OR) for logistic regression, along with 95% confidence intervals (CI). Missing data were handled using complete case analysis. Subgroup analyses were performed to assess the effectiveness of antimicrobial therapy, stratified by disease severity and infection focus. To facilitate subgroup analyses, continuous variables were dichotomized based on the maximal Youden’s index. Two-tailed statistical tests were conducted, and significance was set at *p* < 0.05.

The proportional hazards (PH) assumption of the Cox regression model was assessed by examining Schoenfeld residuals using the *survival* package in R. For covariates that violated the PH assumption, we applied a counting-process approach by restructuring the dataset into a start–stop format with Surv(tstart, tstop, event), thereby allowing the inclusion of time-dependent covariates [37].

## Results

### Demographic characteristics of the study population

A total of 393 patients with critical illness diagnosed with CRAB-BSIs were included (Fig. S1). The mean age of the patients was 66.6 (SD, 16.8) years, and 66.7% were male. Common comorbidities included diabetes mellitus (33.6%), malignancy (24.7%), liver disease (20.6%), and end-stage renal disease (18.6%). The median duration of hospital stay before the BSI onset was 20 (IQR, 11–37) days, and 46.8% of the patients had received antibiotics within the 14 days before BSI onset. The primary causes of infection were pneumonia (42.5%) and catheter-related infections (38.7%) (Table 1).

**Table 1:** Demographic data and laboratory findings of critically ill patients with Carbapenem-resistant *Acinetobacter baumannii* blood stream infection stratified by day-28 survival

Variables	All (*n* = 393)	Survivors (*n* = 171)	Non-survivors (*n* = 222)	*P*-value
Age, mean (SD), years	66.6 (16.8)	64.6 (17.7)	68.0 (15.9)	0.057
Sex, male, n (%)	262 (66.7)	110 (64.3)	152 (68.5)	0.388
BMI, mean (SD), kg/m^2^	23.6 (5.1)	24.2 (5.7)	23.2 (4.5)	0.055
*ICU types, n (%)*				< 0.001
Medical	242 (61.6)	83 (48.5)	159 (71.6)	
Surgical	132 (33.6)	79 (46.2)	53 (23.9)	
Cardiovascular	16 (4.1)	8 (4.7)	8 (3.6)	
Mixed	3 (0.8)	1 (0.6)	2 (0.9)	
*Comorbidities, n (%)*				
Diabetes mellitus	132 (33.6)	50 (29.2)	82 (36.9)	0.109
Malignancy	97 (24.7)	32 (18.7)	65 (29.3)	0.016
Liver disease^a^	81 (20.6)	22 (12.9)	59 (26.6)	0.001
End-stage renal disease	73 (18.6)	33 (19.3)	40 (18.0)	0.746
Chronic lung disease^b^	61 (15.5)	22 (12.9)	39 (17.6)	0.202
Congestive heart failure	50 (12.7)	27 (15.8)	23 (10.4)	0.109
Immunocompromised^c^	53 (13.5)	27 (15.8)	26 (11.7)	0.241
TPN, n (%)	46 (11.7)	16 (9.4)	30 (13.5)	0.204
Antibiotic exposure in the preceding 14 days, n (%)	184 (46.8)	83 (48.5)	101 (45.5)	0.549
*Infection focus, n (%)*				0.133
Pneumonia	167 (42.5)	67 (39.2)	100 (45.0)	
Catheter-related infection	152 (38.7)	68 (39.8)	84 (37.8)	
Intraabdominal infection	34 (8.7)	11 (6.4)	23 (10.4)	
Urinary tract infection	12 (3.1)	8 (4.7)	4 (1.8)	
Soft tissue infection	13 (3.3)	8 (4.7)	5 (2.3)	
Primary bacteremia	10 (2.5)	7 (4.1)	3 (1.4)	
Multiple sites	5 (1.3)	2 (1.2)	3 (1.4)	
Hospital LOS before BSI, median (IQR), days	20 (11–37)	18.5 (10.8–40)	21 (11–36)	0.729
ICU LOS before BSI, median (IQR), days	12 (6–21.5)	12 (6–22.3)	12 (6–20)	0.389
*Antimicrobial susceptibility, n (%)*				
Colistin	282 (71.8)	122 (71.3)	160 (72.1)	0.914
Tigecycline	196 (49.9)	79 (46.2)	117 (52.7)	0.435
Sulbactam	82 (20.9)	54 (31.6)	28 (12.6)	< 0.001
Amikacin	68 (17.3)	37 (21.6)	31 (14.0)	0.137
Gentamycin	42 (10.7)	29 (17.0)	13 (5.9)	0.002
Albumin, median (IQR), g/dL (*n* = 316)	2.7 (2.3–3.0)	2.8 (2.5–3.0)	2.6 (2.2–2.9)	0.001
APACHE II score, median (IQR) (*n* = 365)	23 (18–29)	22 (15–26)	26 (19–32)	< 0.001
SOFA score, median (IQR) (*n* = 351)	10 (7–13)	7 (5–10)	13 (9–16)	< 0.001
Shock, n (%)	174 (44.3)	64 (37.4)	110 (49.5)	0.016
Invasive mechanical ventilation, n (%)	371 (94.4)	163 (95.3)	208 (93.7)	0.486
*Antibiotic regimen*^d^, n (%)				
Carbapenam	169 (43.0)	70 (40.9)	99 (44.6)	0.468
Colistin^f^	163 (41.5)	75 (43.9)	88 (39.6)	0.400
Tigecycline	80 (20.4)	43 (25.1)	37 (16.7)	0.038
Sulbactam	51 (13.0)	32 (18.7)	19 (8.6)	0.003
Aminoglycoside	21 (5.3)	8 (4.7)	13 (5.9)	0.607

^a^Chronic hepatitis or liver cirrhosis.

^b^Asthma, chronic obstructive pulmonary disease, interstitial lung disease, bronchiectasis, or active tuberculosis.

^c^Chronic steroid use (prednisolone 5 mg/day or equivalent > 1 month or > 30 mg/day) or other immunosuppressive therapy for diseases such as connective tissue disease, rheumatic disease, or solid organ transplantation.

^d^Only antibiotics used for more than 2 days and initiated within 3 days of the bloodstream infection onset date were included.

^f^A total of 43 patients (26.4%) received a loading dose of colistin, including 19 in the survivor group and 24 in the non-survivor group.

APACHE II, Acute Physiology and Chronic Health Evaluation II; BMI, body mass index; BSI, bloodstream infection; ICU, intensive care unit; IQR, interquartile range; LOS, length of stay; SD, standard deviation; SOFA, Sequential Organ Failure Assessment; TPN, total parenteral nutrition.

Based on day-28 mortality, patients were stratified into survivors (*n* = 171) and nonsurvivors (*n* = 222). Nonsurvivors had a significantly higher proportion of medical ICU admissions (71.6% vs. 48.5%, *p* < 0.001) and a higher prevalence of malignancy (29.3% vs. 18.7%, *p* = 0.016) and liver disease (26.6% vs. 12.9%, *p* = 0.001) than survivors. In addition, nonsurvivors exhibited significantly higher rates of shock (49.5% vs. 37.4%, *p* = 0.016), higher median APACHE II (26 [IQR, 19–32] vs. 22 [IQR, 15–26], *p* < 0.001) and SOFA (13 [IQR, 9–16] vs. 7 [IQR, 5–10], *p* < 0.001) scores than survivors (Table 1).

When limiting intravenous antibiotics to those initiated within 3 days of CRAB-BSI onset, the most frequently prescribed agents were carbapenem (43%) and colistin (41.5%), followed by tigecycline (20.4%) and sulbactam (13.0%). Among the 163 patients who received colistin, 43 (26.4%) were given a loading dose (Table 1). Based on our antibiotic exposure definition, 45.9% of patients received combination therapy (Table S1), with the most common regimens being carbapenem/colistin, followed by colistin/tigecycline and carbapenem/colistin/tigecycline. Twenty-one patients received regimens involving more than three antibiotics. Detailed antimicrobial regimens are provided in Table S1. Demographic characteristics stratified by antibiotic regimens are presented in Table S2-S4.

### Prognostic factors for day-28 mortality

**Fig. 1:**
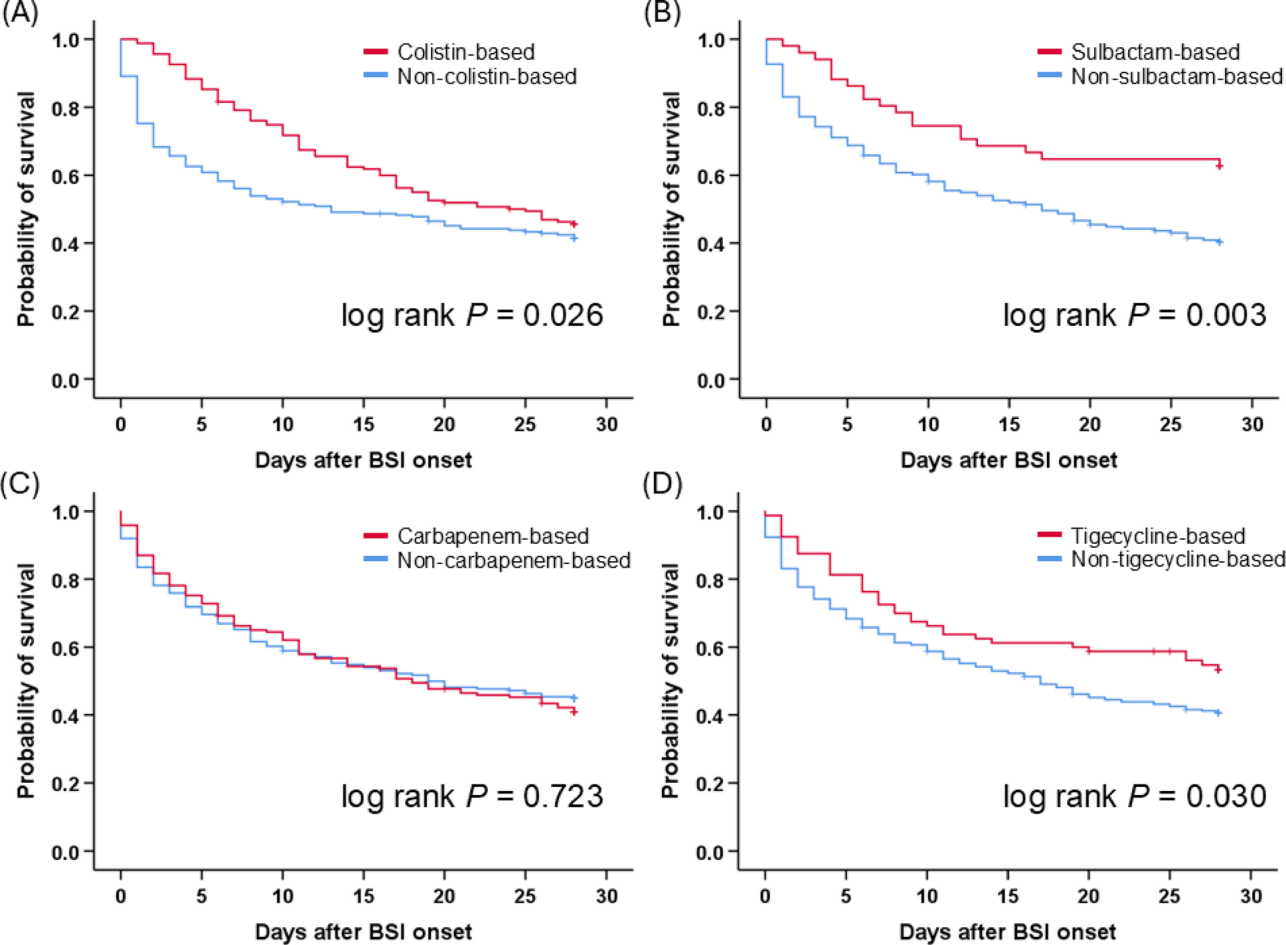
Kaplan-Meier curves for day-28 mortality based on different antibiotic treatment regimens. Survival probabilities are compared between (**A**) colistin-based and non-colistin-based regimens, (**B**) sulbactam-based and non-sulbactam-based regimens, (**C**) carbapenem-based and non-carbapenem-based regimens, and (**D**) tigecycline-based and non-tigecycline-based regimens. Survival differences were assessed using the log-rank test, with corresponding *p*-values displayed in each panel

The day-28 mortality rate of CRAB-BSI in the ICU was 56.5% (Table 1, Table S5). The Kaplan–Meier curves for day-28 mortality across treatment regimens are illustrated in Fig. 1. Patients receiving colistin, sulbactam, or tigecycline exhibited significantly higher cumulative survival rates compared with those not receiving these antibiotics. No difference in the day-28 mortality was found between patients with or without carbapenem treatment.

In the multivariate analysis, higher SOFA scores were independently related to higher day-28 mortality rates in both cohorts. After adjusting for disease severity, comorbidities, and different antimicrobial regimens, colistin-based treatment was independently associated with lower day-28 mortality in both the original (aHR 0.56, 95% CI 0.35–0.88) and time-window bias-adjusted (aHR 0.59, 95% CI 0.37–0.94) cohorts (Table 2). The aHRs for day-28 mortality were 0.75 (95% CI 0.43–1.29) and 0.80 (95% CI 0.45–1.40) for sulbactam-based regimens, and 0.85 (95% CI 0.55–1.32) and 0.93 (95% CI 0.59–1.47) for tigecycline-based regimens.

**Table 2:** Univariate and multivariate analyses of clinical variables associated with day-28 mortality

		Original cohort (*n* = 393)						Time-window bias-adjusted cohort^*a*^ (*n* = 368)					
Variables		Univariate analysis			Multivariate analysis			Univariate analysis			Multivariate analysis		
		HR	95% CI	*P*-value	aHR	95% CI	*P*-value	HR	95% CI	*P*-value	aHR	95% CI	*P*-value
Age		1.01	1.00–1.01	0.116	1.01	1.00–1.02	0.265	1.01	1.00–1.01	0.141	1.01	1.00–1.02	0.235
APACHEII		1.04	1.03–1.06	< 0.001	1.01	0.99–1.03	0.435	1.04	1.02–1.06	< 0.001	1.00	0.98–1.03	0.932
SOFA		1.19	1.15–1.22	< 0.001	1.18	1.13–1.23	< 0.001	1.19	1.16–1.23	< 0.001	1.19	1.14–1.24	< 0.001
Shock		1.47	1.13–1.91	0.004	1.44	1.03–2.03	0.034	1.28	0.97–1.69	0.086	1.13	0.78–1.63	0.516
Pneumonia		1.22	0.93–1.58	0.147				1.26	0.95–1.67	0.102	1.38	0.95–2.00	0.090
Catheter-related infection		0.91	0.69–1.19	0.486				0.99	0.75–1.32	0.969			
Antibiotic exposure		0.98	0.75–1.27	0.860				0.91	0.69–1.20	0.501			
Diabetes mellitus		1.29	0.98–1.69	0.071	1.16	0.81–1.67	0.415	1.28	0.96–1.71	0.095	1.11	0.75–1.64	0.599
Malignancy		1.53	1.15–2.05	0.004	1.46	0.99–2.15	0.055	1.47	1.07–2.00	0.016	1.44	0.94–2.21	0.090
Liver disease		1.67	1.24–2.25	0.001	1.07	0.71–1.63	0.747	1.65	1.20–2.27	0.002	0.96	0.61–1.52	0.878
End-stage renal disease		0.97	0.69–1.36	0.850				0.99	0.69–1.41	0.937			
Albumin level		0.59	0.44–0.79	0.001	1.02	0.73–1.43	0.908	0.56	0.41–0.77	< 0.001	0.93	0.64–1.35	0.700
*Antibiotic regimen* ^b^													
Sulbactam-based		0.50	0.31–0.80	0.004	0.75	0.43–1.29	0.295	0.56	0.35–0.90	0.016	0.80	0.45–1.40	0.427
Colistin-based		0.73	0.56–0.96	0.022	0.56	0.35–0.88	0.012	0.88	0.66–1.16	0.359	0.59	0.37–0.94	0.026
Carbapenam-based		1.05	0.80–1.36	0.733				1.14	0.86–1.51	0.359			
Tigecycline-based		0.68	0.48–0.97	0.032	0.85	0.55–1.32	0.469	0.75	0.52–1.08	0.117	0.93	0.59–1.47	0.758
Aminoglycoside-based		1.09	0.62–1.91	0.764				1.14	0.63–2.04	0.668			

^a^ Patients who died on the BSI onset date were excluded.

^b^ Only antibiotics used for more than 2 days and initiated within 3 days of the bloodstream infection onset date were included.

APACHE II, Acute Physiology and Chronic Health Evaluation II; BSI, bloodstream infection; CI, confidence interval; HR, hazards ratio; SOFA, Sequential Organ Failure Assessment

**Fig. 2:**
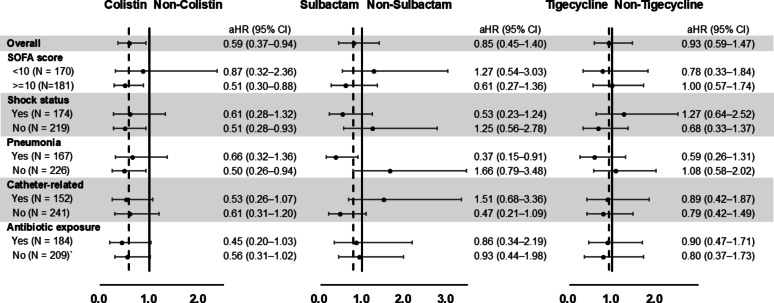
Forest plot of adjusted hazard ratios for day-28 mortality in different subgroups, comparing different antibiotic regimens. Analyses were adjusted for age, malignancy, liver disease, diabetes mellitus, albumin level, APACHE II score, SOFA score, shock status, and antibiotic regimen. aHR, adjusted hazard ratio; APACHE II, Acute Physiology and Chronic Health Evaluation II; CI, confidence interval; SOFA, Sequential Organ Failure Assessment.

A forest plot was generated to demonstrate subgroup analyses across different antimicrobial regimens. Colistin-based therapy was associated with a lower risk of day-28 mortality among patients with a SOFA score ≥ 10 (aHR 0.51, 95% CI 0.30–0.88) (Fig. 2). Among patients with pneumonia, colistin-based therapy did not improve day-28 mortality (aHR 0.66, 95% CI 0.32–1.36), whereas sulbactam-based therapy provided survival benefit (aHR 0.37, 95% CI 0.15–0.91).

### Clinical and microbiological outcomes

The clinical and microbiological outcomes across the different patient subgroups are detailed in Table S5. Patients with comorbid malignancy or liver disease exhibited significantly higher clinical failure rates and lower microbiological eradication rates on day 14, and higher day-28 and in-hospital mortality rates. Notably, pneumonia-related CRAB-BSIs demonstrated a significantly higher clinical failure rate (64.1%) and a lower microbiological eradication rate (32.3%) on day 28.

Table 3 presents the adjusted OR (aOR) for the clinical and microbiological outcomes associated with different antimicrobial regimens, derived from the logistic regression analysis. In the original cohort (*N* = 393), colistin-based therapy significantly improved the microbiological eradication rates on days 7 (aOR 2.19, 95% CI 1.18–4.07) and 14 (aOR 2.33, 95% CI 1.22–4.47), while reduced the clinical failure rates on days 7 (aOR 0.31, 95% CI 0.16–0.57), 14 (aOR 0.41, 95% CI 0.22–0.78), and 28 (aOR 0.42, 95% CI 0.22–0.79). The benefit of colistin-based therapy remained observable in the adjusted cohort (*N* = 368).

**Table 3:** Logistic regression for assessing the clinical and Microbiological outcomes associated with different antibiotic regimens

		Original cohort (*n* = 393)			Time-window bias-adjusted cohort^a^ (*n* = 368)		
		Colistin-based aOR (95% CI)^b^	Sulbactam-based aOR (95% CI)^c^	Tigecycline-based aOR (95% CI)^d^	Colistin-based aOR (95% CI)^b^	Sulbactam-based aOR (95% CI)^c^	Tigecycline-based aOR (95% CI)^d^
*Microbiology eradication*							
Day 7		2.19 (1.18–4.07)	1.60 (0.70–3.67)	0.96 (0.48–1.92)	1.92 (1.03–3.57)	1.49 (0.66–3.40)	0.91 (0.46–1.82)
Day 14		2.33 (1.22–4.47)	2.15 (0.87–5.35)	0.64 (0.31–1.31)	1.98 (1.03–3.82)	1.95 (0.79–4.82)	0.60 (0.29–1.22)
Day 28		1.70 (0.91–3.20)	1.84 (0.79–4.29)	0.94 (0.46–1.91)	1.51 (0.80–2.85)	1.73 (0.75–4.00)	0.90 (0.45–1.82)
*Clinical failure*							
Day 7		0.31 (0.16–0.57)	0.97 (0.42–2.27)	0.88 (0.44–1.77)	0.36 (0.19–0.68)	1.06 (0.46–2.45)	0.96 (0.48–1.92)
Day 14		0.41 (0.22–0.78)	0.63 (0.26–1.54)	1.05 (0.52–2.12)	0.49 (0.26–0.93)	0.70 (0.29–1.70)	1.14 (0.56–2.33)
Day 28		0.42 (0.22–0.79)	1.08 (0.46–2.53)	0.90 (0.44–1.83)	0.48 (0.25–0.91)	1.14 (0.49–2.67)	0.96 (0.47–1.94)

^a^ Patients who died on the BSI onset date were excluded.

^b^ Adjusted by age, malignancy, liver disease, diabetes mellitus, albumin level, APACHE II score, SOFA score, shock status, sulbactam and tigecycline.

^c^ Adjusted by age, malignancy, liver disease, diabetes mellitus, albumin level, APACHE II score, SOFA score, shock status, colistin and tigecycline.

^d^ Adjusted by age, malignancy, liver disease, diabetes mellitus, albumin level, APACHE II score, SOFA score, shock status, colistin and sulbactam.

APACHE II, Acute Physiology and Chronic Health Evaluation II; BSI, bloodstream infection; CI, confidence interval, OR: odds ratio; SOFA, Sequential Organ Failure Assessment

Neither sulbactam- nor tigecycline-based therapies demonstrated significant effects on the microbiological eradication or clinical failure rates.

## Discussion

This multicenter investigation evaluates CRAB-BSI outcomes in the ICU and examines the role of different antimicrobial regimens. The day-28 mortality rate was high (56.5%) in critically ill patients with CRAB-BSI. Colistin-based therapy was associated with reduced day-28 mortality, higher microbiological eradication, and lower clinical failure rates. Subgroup analyses revealed that colistin-based therapy offered significant survival benefits among patients with higher SOFA scores and non-pneumonia infections. In contrast, sulbactam-based therapy reduced mortality in patients with pneumonia-related CRAB-BSIs. These findings provide important insights into the treatment of CRAB-BSIs using the best available therapies when novel antibiotics are not accessible.

CRAB-BSIs showed a high mortality rate in this study, which is consistent with the previous literature. A single-center ICU study conducted in Korea (2008–2009) reported a 30-day mortality rate of 79.8% among 106 patients with critical illness and CRAB-BSIs [33]. In addition, a multicenter Korean analysis (2012–2015) reported a day-28 mortality rate of 69.8% in 143 cases, including 117 ICU admissions [38]. More recently, a Greek ICU cohort study involving 172 patients with critical illness and CRAB-BSIs demonstrated a day-28 mortality rate of 53% [34]. To the best of our knowledge, the present study represents the largest multicenter investigation of CRAB-BSI in the ICU to date, further underscoring the significance of its mortality burden.

In this study, colistin-based therapy was associated with significantly higher microbiological eradication, lower clinical failure, and reduced mortality rates among patients with CRAB-BSIs. This observation aligns with the results of previous studies indicating that early intravenous colistin therapy improves day-28 mortality in patients with CRAB-BSIs [32]. However, colistin-based therapy did not yield significant survival benefits among patients with pneumonia-related CRAB-BSIs. This finding may be explained by the suboptimal penetration and activity of intravenous polymyxins within the pulmonary epithelial lining fluid [39, 40]. In such cases, combination therapy with additional antibiotics should be considered. Previous studies have demonstrated that combining colistin with high-dose ampicillin-sulbactam results in improved clinical responses in CRAB-associated ventilator-associated pneumonia [41].

The Infectious Diseases Society of America (IDSA) suggests the use of polymyxin B—a drug associated with lower renal toxicity than colistin [42] —as an option for combination therapy in the treatment of CRAB infections, particularly when novel antibiotics are not available [18]. However, polymyxin B was unavailable in Taiwan during the study period. Nonetheless, current evidence indicates comparable therapeutic efficacy between polymyxin B and colistin [43, 44]. Thus, polymyxin B may offer similar efficacy to colistin in treating CRAB-BSIs while mitigating concerns related to renal toxicity and should be used in treating CRAB-BSIs, if available.

Sulbactam has long been considered the backbone of treatment for CRAB. The IDSA and European Society of Clinical Microbiology and Infectious Diseases guidelines recommend the use of high-dose ampicillin-sulbactam (total daily dose of 9 g sulbactam component) combined with at least one additional antimicrobial agent for CRAB infections [18, 31]. In our cohort, sulbactam therapy was associated with higher cumulative survival in patients with pneumonia-related BSI. A recent meta-analysis indicated that therapeutic regimens combining high-dose sulbactam with additional antimicrobial agents were most effective in reducing mortality among patients with critical illness and CRAB infections [29]; notably, most included studies focused primarily on pneumonia cases. Because pneumonia is a major source of CRAB-BSIs, adequate drug concentrations must be achieved in the lung tissue and bloodstream. Even in the case of sulbactam resistance, its pharmacokinetic properties enable high-dose regimens to overcome resistance mechanisms [45]. Although sulbactam was prescribed in only 13% of the cases in this study—potentially contributing to the lack of significant treatment outcomes—our findings remain consistent with current evidence supporting the use of sulbactam-based therapy for pneumonia-related CRAB-BSIs.

Carbapenem-based therapy, despite being the most frequently prescribed antibiotic, did not provide a survival benefit for patients with CRAB-BSI in this study. This result aligns with the findings of two large randomized controlled trials demonstrating that adding meropenem to the colistin regimen did not improve survival in patients with CRAB infections [46, 47]. The prevalent use of carbapenems in our cohort may reflect adherence to local guidelines during the study period, which recommended combination therapy with colistin and meropenem for CRAB infections [30]. However, updated international guidelines advise against carbapenem combinations for treating CRAB infections [31], except when combined with sulbactam-durlobactam [18]. This study provides real-world evidence suggesting that carbapenem-based treatments may be less favorable in the management of CRAB-BSIs.

This study has several limitations. First, given the retrospective design, clinical complexity of patients with critical illness, and diversity of antimicrobial regimens, detailed antibiotic dosages or side effects, such as colistin-associated nephrotoxicity, were not reported. Antibiotic dosages were adjusted by clinicians according to the available guidelines and each patient’s renal function. Second, the sulbactam-based group includes a relatively small number of patients; therefore, our findings need to be validated by studies with larger case numbers. Third, defining antibiotic exposure based on at least 2 days of use may have introduced immortal time bias, as patients had to survive long enough to meet this threshold. Fourth, due to frequent regimen adjustments in ICU settings, exposure misclassification may have occurred, particularly in cases involving overlapping or switched therapies. Fifth, the definition of clinical and microbiological outcomes across different infection foci is subjective and inherently complex—particularly in cases of bacteremic pneumonia, where radiographic improvement and microbiological clearance of respiratory pathogens should also be considered. In this study, however, treatment outcomes were defined based on BSI-related parameters, which may not fully capture the clinical response to pneumonia treatment. Finally, this study focused only on the prognostic outcomes associated with conventional antibiotics. Therefore, the findings may not be generalizable to novel antibiotics used for treating CRAB-BSI in patients with critical illness. Nevertheless, these findings contribute valuable evidence to guide optimal patient management with existing therapies until newer antibiotics are more readily available.

## Conclusions

CRAB-BSIs were associated with high mortality in patients with critical illness. A higher SOFA score independently predicted day-28 mortality. In the circumstance where novel antibiotics are not available, colistin-based therapy was associated with improved treatment outcomes, whereas carbapenem-based therapy did not show survival advantage. For patients with pneumonia-related CRAB-BSIs, sulbactam-based therapy significantly reduced the mortality risk. More prospective studies are warranted to confirm these findings and enhance therapeutic strategies using conventional antibiotics and novel agents to effectively manage this challenging infection.

## Supplementary Information

Additional file 1.

## Data Availability

The data of this study are available on request from the corresponding author.

## Abbreviations

APACHE II: Acute Physiologic Assessment and Chronic Health Evaluation II
BSI: Bloodstream infection
CI: Confidence interval
CRAB: Carbapenem-Resistant* Acinetobacter baumannii*
CVC: Central venous catheter
HR: Hazard ratio
ICU: Intensive care unit
IDSA: Infectious Diseases Society of America
IQR: Interquartile range
IRB: Institutional review board
OR: Odds ratio
SD: Standard deviation
SOFA: Sequential organ failure assessment
