# Supplementary material for: Outcomes of carbapenem-resistant Acinetobacter baumannii bloodstream infections in intensive care units and prognostic effect of different antimicrobial regimens
Source: Ann Intensive Care. 2025 Oct 18;15:162. doi: 10.1186/s13613-025-01580-7 (PMC12534662; doi:10.1186/s13613-025-01580-7)
Supplement: Supplementary file 1 — Additional file 1. [file 13613_2025_1580_MOESM1_ESM.docx]

**Supplementary Materials**

**Definitions for specific types of infections:**

***Pneumonia*** was defined by the appearance of a new lung infiltrate on chest imaging, combined with at least two clinical symptoms such as the new onset of fever, purulent sputum, leukocytosis, and a decrease in oxygenation.

***Urinary Tract Infection*** was defined as having at least one of the following signs or symptoms in addition to pyuria and a positive urine culture: fever, urgency, frequency, dysuria, or suprapubic tenderness, with no other recognized cause.

***Soft Tissue Infection*** was defined as having at least two of the following signs or symptoms: localized pain, tenderness, swelling, erythema, or heat, with organisms identified from aspirate or drainage from the affected site.

***Intraabdominal Infection*** was defined as having at least two of the following signs or symptoms: fever, hypotension, nausea, vomiting, abdominal pain or tenderness, elevated transaminase levels, or jaundice, with at least one of the following: organisms seen on Gram stain or identified from intraabdominal fluid or tissue obtained during an invasive procedure or from an aseptically-placed drain, or imaging test evidence suggestive of infection, supported by clinical correlation.

***Catheter-related Infection*** was defined in patients with an indwelling central venous catheter and no clear source of BSI.

***Primary bacteremia*** was defined by the absence of an identifiable source of infection that cultured the same organism(s) as found in the blood and the absence of a central vascular catheter.

**Table S1.** Detailed antibiotic regimens used in this study. Intravenous antibiotics administered for at least 2 days and initiated within 3 days of the carbapenem-resistant *Acinetobacter baumannii* bloodstream infection onset date were recorded. An asterisk (*) and a caret (^) indicate combination therapy, defined as the concurrent use of at least two intravenous antibiotics with an overlap of at least 2 days.

| **Regimen** | **Case number (n = 294)** | **Colistin based**  **(n = 163)** | **Sulbactam based**  **(n = 51)** | **Tigecycline based**  **(n = 80)** |
| --- | --- | --- | --- | --- |
| Colistin | 40 | 40 | 0 | 0 |
| Colistin + Amikacin | 2 | 2 | 0 | 0 |
| Colistin* + Amikacin* | 1^a^ | 1^a^ | 0 | 0 |
| Colistin + Carbapenam | 8 | 8 | 0 | 0 |
| Colistin* + Carbapenam* | 53^a^ | 53^a^ | 0 | 0 |
| Colistin + Sulbactam | 1 | 1 | 1 | 0 |
| Colistin* + Sulbactam* | 8^a^ | 8^a^ | 8^a^ | 0 |
| Colistin* + Tigecycline* | 15^a^ | 15^a^ | 0 | 15^a^ |
| Colistin* + Carbapenam* + Amikacin | 1^a^ | 1^a^ | 0 | 0 |
| Colistin* + Carbapenam* + Amikacin* | 1^b^ | 1^b^ | 0 | 0 |
| Colistin* + Carbapenam*^ + Amikacin^ | 1^a^ | 1^a^ | 0 | 0 |
| Colistin + Carbapenam + Tigecycline | 1 | 1 | 0 | 1 |
| Colistin + Carbapenam* + Tigecycline* | 1^a^ | 1^a^ | 0 | 1^a^ |
| Colistin* + Carbapenam* + Tigecycline | 2^a^ | 2^a^ | 0 | 2^a^ |
| Colistin* + Carbapenam* + Tigecycline* | 11^b^ | 11^b^ | 0 | 11^b^ |
| Colistin* + Carbapenam*^ + Tigecycline^ | 1^a^ | 1^a^ | 0 | 1^a^ |
| Colistin*^ + Carbapenam* + Tigecycline^ | 1^a^ | 1^a^ | 0 | 1^a^ |
| Colistin* + Carbapenam* + Sulbactam | 1^a^ | 1^a^ | 1^a^ | 0 |
| Colistin* + Sulbactam* + Carbapenam | 2^a^ | 2^a^ | 2^a^ | 0 |
| Colistin* + Sulbactam* + Carbapenam* | 2^b^ | 2^b^ | 2^b^ | 0 |
| Colistin* + Sulbactam* + Tigecycline* | 1^b^ | 1^b^ | 1^b^ | 1^b^ |
| Colistin*^ + Sulbactam* + Tigecycline^ | 1^a^ | 1^a^ | 1^a^ | 1^a^ |
| Colistin* + Tigecycline* + Carbapenam | 1^a^ | 1^a^ | 0 | 1^a^ |
| Colistin* + Tigecycline*^ + Carbapenam^ | 1^a^ | 1^a^ | 0 | 1^a^ |
| Colistin* + Tigecycline* + Sulbactam | 1^a^ | 1^a^ | 1^a^ | 1^a^ |
| Colistin* + Carbapenam* + Sulbactam^ + Amikacin^ | 2^a^ | 2^a^ | 2^a^ | 0 |
| Colistin* + Sulbactam* + Carbapenam* + Tigecycline | 1^b^ | 1^b^ | 1^b^ | 1^b^ |
| Colistin* + Sulbactam* + Carbapenam* + Tigecycline* | 1^b^ | 1^b^ | 1^b^ | 1^b^ |
| Colistin* + Tigecycline*^ + Carbapenam^ + Gentamycin^ | 1^b^ | 1^b^ | 0 | 1^b^ |
| Sulbactam | 16 | 0 | 16 | 0 |
| Sulbactam + Amikacin | 1 | 0 | 1 | 0 |
| Sulbactam + Carbapenam | 4 | 0 | 4 | 0 |
| Sulbactam* + Carbapenam* | 4^a^ | 0 | 4^a^ | 0 |
| Sulbactam + Tigecycline | 1 | 0 | 1 | 1 |
| Sulbactam* + Tigecycline* | 2^a^ | 0 | 2^a^ | 2^a^ |
| Sulbactam + Carbapenam* + Tigecycline* | 1^a^ | 0 | 1^a^ | 1^a^ |
| Sulbactam* + Carbapenam* + Tigecycline* | 1^b^ | 0 | 1^b^ | 1^b^ |
| Tigecycline | 15 | 0 | 0 | 15 |
| Tigecycline* + Amikacin* | 2^a^ | 0 | 0 | 2^a^ |
| Tigecycline + Carbapenam | 4 | 0 | 0 | 4 |
| Tigecycline* + Carbapenam* | 12^a^ | 0 | 0 | 12^a^ |
| Tigecycline* + Carbapenam* + Amikacin* | 2^b^ | 0 | 0 | 2^b^ |
| Combination treatment, n (%) | 135 (45.9) | 111 (68.1) | 28 (54.9) | 59 (73.8) |
| ^a^ Combination with two antibiotics  ^b^ Combination with three or more antibiotics | | | | |

**Table S2.** Demographic characteristics of critically ill patients with carbapenem-resistant *Acinetobacter baumannii* bloodstream infection, stratified by colistin-based versus non–colistin-based therapy.

| **Variables** | **All**  **(n = 393)** | **Colistin-based**  **(n = 163)** | **Non–colistin-based**  **(n = 230)** | ***P*-value** |  |
| --- | --- | --- | --- | --- | --- |
| Age, mean (SD), years | 66.6 (16.8) | 64.0 (18.1) | 68.7 (15.9) | 0.009 |  |
| Sex, male, n (%) | 262 (66.7) | 113 (69.3) | 149 (64.8) | 0.405 |  |
| BMI, mean (SD), kg/m^2^ | 23.6 (5.1) | 23.8 (5.7) | 23.6 (4.5) | 0.699 |  |
| ICU types, n (%) |  |  |  | 0.364 |  |
| Medical | 242 (61.6) | 95 (58.3) | 147 (63.9) |  |  |
| Surgical | 132 (33.6) | 61 (37.4) | 71 (30.9) |  |  |
| Cardiovascular | 16 (4.1) | 5 (3.1) | 11 (4.8) |  |  |
| Mixed | 3 (0.8) | 2 (1.2) | 1 (0.4) |  |  |
| Comorbidities, n (%) |  |  |  |  |  |
| Diabetes mellitus | 132 (33.6) | 33 (20.2) | 99 (43.0%) | <0.001 |  |
| Malignancy | | 97 (24.7) | 37 (22.7) | 60 (26.1) | 0.517 |
| Liver disease^a^ | 81 (20.6) | 37 (22.7) | 44 (19.1) | 0.462 |  |
| End-stage renal disease | 73 (18.6) | 31 (19.0) | 42 (18.3) | 0.953 |  |
| Chronic lung disease^b^ | 61 (15.5) | 24 (14.7) | 37 (16.1) | 0.821 |  |
| Congestive heart failure | 50 (12.7) | 20 (12.3) | 30 (13.0) | 0.942 |  |
| Immunocompromised^c^ | 53 (13.5) | 19 (11.7) | 34 (14.8) | 0.457 |  |
| TPN, n (%) | 46 (11.7) | 23 (14.1) | 23 (10.0) | 0.276 |  |
| Antibiotic exposure in the preceding 14 days, n (%) | 184 (46.8) | 62 (38.0) | 122 (53.0) | 0.005 |  |
| Infection focus, n (%) |  |  |  | 0.215 |  |
| Pneumonia | 167 (42.5) | 73 (44.8) | 94 (40.9) |  |  |
| Catheter-related infection | 152 (38.7) | 69 (42.3) | 83 (36.1) |  |  |
| Intraabdominal infection | 34 (8.7) | 8 (4.9) | 26 (11.3) |  |  |
| Urinary tract infection | 12 (3.1) | 5 (3.1) | 7 (3.0) |  |  |
| Soft tissue infection | 13 (3.3) | 4 (2.5) | 9 (3.9) |  |  |
| Primary bacteremia | 10 (2.5) | 2 (1.2) | 8 (3.5) |  |  |
| Multiple sites | 5 (1.3) | 2 (1.2) | 3 (1.3) |  |  |
| Hospital LOS before BSI, median (IQR), days | 20 (11–37) | 22 (12–38) | 18 (10–37) | 0.389 |  |
| ICU LOS before BSI, median (IQR), days | 12 (6–21.5) | 13 (7–23) | 12 (5–20) | 0.016 |  |
| Antimicrobial susceptibility, n (%) |  |  |  |  |  |
| Colistin | 282 (71.8) | 117 (71.8) | 165 (71.7) | 0.878 |  |
| Tigecycline | 196 (49.9) |  |  |  |  |
| Sulbactam | 82 (20.9) | 31 (19.0) | 51 (22.2) | 0.067 |  |
| Amikacin | 68 (17.3) | 19 (11.7) | 49 (21.3) | 0.004 |  |
| Gentamycin | 42 (10.7) | 14 (8.6) | 28 (12.2) | 0.357 |  |
| Albumin, median (IQR), g/dL (n = 316) | 2.7 (2.3–3.0) | 2.7 (2.4–3.0) | 2.6 (2.2–2.9) | 0.230 |  |
| APACHE II score, median (IQR) (n = 365) | 23 (18–29) | 22.0 (17–28) | 24 (19–31) | 0.011 |  |
| SOFA score, median (IQR) (n = 351) | 10 (7–13) | 10 (7–14) | 10 (6.5–13) | 0.527 |  |
| Shock, n (%) | 174 (44.3) | 63 (38.7) | 111 (48.3) | 0.074 |  |
| Invasive mechanical ventilation, n (%) | 371 (94.4) | 158 (96.9) | 213 (92.6) | 0.106 |  |
| Antibiotic regimen^d^, n (%) |  |  |  |  |  |
| Carbapenam | 169 (43.0) | 87 (53.4) | 82 (35.7) | <0.001 |  |
| Tigecycline | 80 (20.4) | 40 (24.5) | 40 (17.4) | 0.108 |  |
| Sulbactam | 51 (13.0) | 21 (12.9) | 30 (13.0) | >0.999 |  |
| Aminoglycoside | 21 (5.3) | 9 (5.5) | 12 (5.2) | >0.999 |  |
| ^a^ Chronic hepatitis or liver cirrhosis.  ^b^ Asthma, chronic obstructive pulmonary disease, interstitial lung disease, bronchiectasis, or active tuberculosis.  ^c^ Chronic steroid use (prednisolone 5 mg/day or equivalent > 1 month or > 30 mg/day) or other immunosuppressive therapy for diseases such as connective tissue disease, rheumatic disease, or solid organ transplantation.  ^d^ Only antibiotics used for more than 2 days and initiated within 3 days of the bloodstream infection onset date were included. | | | | |  |

**Table S3.** Demographic characteristics of critically ill patients with carbapenem-resistant *Acinetobacter baumannii* bloodstream infection, stratified by tigecycline-based versus non–tigecycline-based therapy.

| **Variables** | **All**  **(n = 393)** | **Tigecycline-based**  **(n = 80)** | **Non–Tigecycline-based**  **(n = 313)** | ***P*-value** |  |
| --- | --- | --- | --- | --- | --- |
| Age, mean (SD), years | 66.6 (16.8) | 64.6 (16.5) | 67.3 (17.0) | 0.203 |  |
| Sex, male, n (%) | 262 (66.7) | 48 (60.0) | 214 (68.4) | 0.199 |  |
| BMI, mean (SD), kg/m^2^ | 23.6 (5.1) | 23.6 (5.2) | 23.7 (5.1) | 0.869 |  |
| ICU types, n (%) |  |  |  | 0.086 |  |
| Medical | 242 (61.6) | 43 (53.8) | 199 (63.6) |  |  |
| Surgical | 132 (33.6) | 30 (37.5) | 102 (32.6) |  |  |
| Cardiovascular | 16 (4.1) | 5 (6.3) | 11 (3.5) |  |  |
| Mixed | 3 (0.8) | 2 (2.5) | 1 (0.3) |  |  |
| Comorbidities, n (%) |  |  |  |  |  |
| Diabetes mellitus | 132 (33.6) | 22 (27.5) | 110 (35.1) | 0.246 |  |
| Malignancy | | 97 (24.7) | 20 (25.0) | 77 (24.6) | >0.999 |
| Liver disease^a^ | 81 (20.6) | 10 (12.5) | 71 (22.7) | 0.064 |  |
| End-stage renal disease | 73 (18.6) | 11 (13.8) | 62 (19.8) | 0.279 |  |
| Chronic lung disease^b^ | 61 (15.5) | 12 (15.0) | 49 (15.7) | >0.999 |  |
| Congestive heart failure | 50 (12.7) | 14 (17.5) | 36 (11.5) | 0.212 |  |
| Immunocompromised^c^ | 53 (13.5) | 14 (17.5) | 39 (12.5) | 0.320 |  |
| TPN, n (%) | 46 (11.7) | 5 (6.3) | 41 (13.1) | 0.132 |  |
| Antibiotic exposure in the preceding 14 days, n (%) | 184 (46.8) | 49 (61.3) | 135 (43.1) | 0.006 |  |
| Infection focus, n (%) |  |  |  | 0.132 |  |
| Pneumonia | 167 (42.5) | 28 (35.0) | 139 (44.4) |  |  |
| Catheter-related infection | 152 (38.7) | 35 (43.8) | 117 (37.4) |  |  |
| Intraabdominal infection | 34 (8.7) | 8 (10.0) | 26 (8.3) |  |  |
| Urinary tract infection | 12 (3.1) | 3 (3.8) | 9 (2.9) |  |  |
| Soft tissue infection | 13 (3.3) | 3 (3.8) | 10 (3.2) |  |  |
| Primary bacteremia | 10 (2.5) | 0 (0.0) | 10 (3.2) |  |  |
| Multiple sites | 5 (1.3) | 3 (3.8) | 2 (0.6) |  |  |
| Hospital LOS before BSI, median (IQR), days | 20 (11–37) | 22 (10.5–39.5) | 20 (11–36) | 0.623 |  |
| ICU LOS before BSI, median (IQR), days | 12 (6–21.5) | 12 (7–22) | 12 (6–21) | 0.761 |  |
| Antimicrobial susceptibility, n (%) |  |  |  |  |  |
| Colistin | 282 (71.8) | 49 (61.3) | 233 (74.4) | 0.051 |  |
| Tigecycline | 196 (49.9) | 48 (60.0) | 148 (47.3) | 0.003 |  |
| Sulbactam | 82 (20.9) | 13 (16.3) | 69 (22.0) | 0.462 |  |
| Amikacin | 68 (17.3) | 6 (7.5) | 62 (19.8) | 0.017 |  |
| Gentamycin | 42 (10.7) | 6 (7.5) | 36 (11.5) | 0.295 |  |
| Albumin, median (IQR), g/dL (n = 316) | 2.7 (2.3–3.0) | 2.6 (2.2–3.0) | 2.7 (2.3–3.0) | 0.604 |  |
| APACHE II score, median (IQR) (n = 365) | 23 (18–29) | 21 (17–25.5) | 24 (18–30) | 0.003 |  |
| SOFA score, median (IQR) (n = 351) | 10 (7–13) | 10 (7–12) | 10 (7–14) | 0.153 |  |
| Shock, n (%) | 174 (44.3) | 39 (48.8) | 135 (43.1) | 0.437 |  |
| Invasive mechanical ventilation, n (%) | 371 (94.4) | 72 (90.0) | 299 (95.5) | 0.100 |  |
| Antibiotic regimen^d^, n (%) |  |  |  |  |  |
| Carbapenam | 169 (43.0) | 34 (42.5) | 135 (43.1) | >0.999 |  |
| Colistin | 163 (41.5) | 40 (50.0) | 123 (39.3) | 0.108 |  |
| Sulbactam | 51 (13.0) | 10 (12.5) | 41 (13.1) | >0.999 |  |
| Aminoglycoside | 21 (5.3) | 5 (6.3) | 16 (5.1) | 0.900 |  |
| ^a^ Chronic hepatitis or liver cirrhosis.  ^b^ Asthma, chronic obstructive pulmonary disease, interstitial lung disease, bronchiectasis, or active tuberculosis.  ^c^ Chronic steroid use (prednisolone 5 mg/day or equivalent > 1 month or > 30 mg/day) or other immunosuppressive therapy for diseases such as connective tissue disease, rheumatic disease, or solid organ transplantation.  ^d^ Only antibiotics used for more than 2 days and initiated within 3 days of the bloodstream infection onset date were included. | | | | |  |

**Table S4.** Demographic characteristics of critically ill patients with carbapenem-resistant *Acinetobacter baumannii* bloodstream infection, stratified by sulbactam-based versus non–sulbactam-based therapy.

| **Variables** | **All**  **(n = 393)** | **Sulbactam-based**  **(n = 51)** | **Non–Sulbactam-based**  **(n = 342)** | ***P*-value** |  |
| --- | --- | --- | --- | --- | --- |
| Age, mean (SD), years | 66.6 (16.8) | 66.9 (18.9) | 66.7 (16.7) | 0.939 |  |
| Sex, male, n (%) | 262 (66.7) | 39 (76.5) | 223 (65.2) | 0.152 |  |
| BMI, mean (SD), kg/m^2^ | 23.6 (5.1) | 23.6 (4.9) | 23.7 (5.1) | 0.939 |  |
| ICU types, n (%) |  |  |  | 0.311 |  |
| Medical | 242 (61.6) | 26 (51.0) | 216 (63.2) |  |  |
| Surgical | 132 (33.6) | 22 (43.1) | 110 (32.2) |  |  |
| Cardiovascular | 16 (4.1) | 3 (5.9) | 13 (3.8) |  |  |
| Mixed | 3 (0.8) | 0 (0.0) | 3 (0.9) |  |  |
| Comorbidities, n (%) |  |  |  |  |  |
| Diabetes mellitus | 132 (33.6) | 17 (33.3) | 115 (33.6) | >0.999 |  |
| Malignancy | | 97 (24.7) | 9 (17.6) | 88 (25.7) | 0.282 |
| Liver disease^a^ | 81 (20.6) | 4 (7.8) | 77 (22.5) | 0.026 |  |
| End-stage renal disease | 73 (18.6) | 7 (13.7) | 66 (19.3) | 0.446 |  |
| Chronic lung disease^b^ | 61 (15.5) | 5 (9.8) | 56 (16.4) | 0.317 |  |
| Congestive heart failure | 50 (12.7) | 7 (13.7) | 43 (12.6) | 0.996 |  |
| Immunocompromised^c^ | 53 (13.5) | 5 (9.8) | 48 (14.0) | 0.545 |  |
| TPN, n (%) | 46 (11.7) | 5 (9.8) | 41 (12.0) | 0.826 |  |
| Antibiotic exposure in the preceding 14 days, n (%) | 184 (46.8) | 22 (43.1) | 162 (47.4) | 0.679 |  |
| Infection focus, n (%) |  |  |  | 0.616 |  |
| Pneumonia | 167 (42.5) | 24 (47.1) | 143 (41.8) |  |  |
| Catheter-related infection | 152 (38.7) | 21 (41.2) | 131 (38.3) |  |  |
| Intraabdominal infection | 34 (8.7) | 1 (2.0) | 33 (9.6) |  |  |
| Urinary tract infection | 12 (3.1) | 2 (3.9) | 10 (2.9) |  |  |
| Soft tissue infection | 13 (3.3) | 2 (3.9) | 11 (3.2) |  |  |
| Primary bacteremia | 10 (2.5) | 1 (2.0) | 9 (2.6) |  |  |
| Multiple sites | 5 (1.3) | 0 (0.0) | 5 (1.5) |  |  |
| Hospital LOS before BSI, median (IQR), days | 20 (11–37) | 20.0 (14–37) | 20.0 (11–37) | 0.339 |  |
| ICU LOS before BSI, median (IQR), days | 12 (6–21.5) | 15.0 (6–19) | 12.0 (6–22) | 0.340 |  |
| Antimicrobial susceptibility, n (%) |  |  |  |  |  |
| Colistin | 282 (71.8) | 39 (76.5) | 243 (71.1) | 0.464 |  |
| Tigecycline | 196 (49.9) | 25 (49.0) | 171 (50.0) | 0.754 |  |
| Sulbactam | 82 (20.9) | 20 (39.2) | 62 (18.1) | 0.002 |  |
| Amikacin | 68 (17.3) | 13 (25.5) | 55 (16.1) | 0.113 |  |
| Gentamycin | 42 (10.7) | 6 (11.8) | 36 (10.5) | 0.306 |  |
| Albumin, median (IQR), g/dL (n = 316) | 2.7 (2.3–3.0) | 2.7 (2.4–3.0) | 2.6 (2.3–3.0) | 0.273 |  |
| APACHE II score, median (IQR) (n = 365) | 23 (18–29) | 20 (14–26) | 24 (18–30) | 0.002 |  |
| SOFA score, median (IQR) (n = 351) | 10 (7–13) | 8 (5–10) | 10 (7–14) | <0.001 |  |
| Shock, n (%) | 174 (44.3) | 24 (47.1) | 150 (43.9) | 0.781 |  |
| Invasive mechanical ventilation, n (%) | 371 (94.4) | 46 (90.2) | 325 (95.0) | 0.283 |  |
| Antibiotic regimen^d^, n (%) |  |  |  |  |  |
| Carbapenam | 169 (43.0) | 17 (33.3) | 152 (44.4) | 0.179 |  |
| Tigecycline | 80 (20.4) | 10 (19.6) | 70 (20.5) | >0.999 |  |
| Colistin | 163 (41.5) | 21 (41.2) | 142 (41.5) | >0.999 |  |
| Aminoglycoside | 21 (5.3) | 3 (5.9) | 18 (5.3) | >0.999 |  |
| ^a^ Chronic hepatitis or liver cirrhosis.  ^b^ Asthma, chronic obstructive pulmonary disease, interstitial lung disease, bronchiectasis, or active tuberculosis.  ^c^ Chronic steroid use (prednisolone 5 mg/day or equivalent > 1 month or > 30 mg/day) or other immunosuppressive therapy for diseases such as connective tissue disease, rheumatic disease, or solid organ transplantation.  ^d^ Only antibiotics used for more than 2 days and initiated within 3 days of the bloodstream infection onset date were included. | | | | |  |

**Table S5.** Clinical and microbiological outcomes among different subgroups.

| **Variables** | **Day-28 mortality** | **In-hospital mortality** | **Clinical failure**  **Day_7** | **Clinical failure**  **Day_14** | **Clinical failure**  **Day_28** | **Microbiologic eradication**  **Day_7** | **Microbiologic eradication**  **Day_14** | **Microbiologic eradication**  **Day_28** |
| --- | --- | --- | --- | --- | --- | --- | --- | --- |
| Overall (n = 393) | 222 (56.5%) | 265 (67.4%) | 188 (47.8%) | 203 (51.7%) | 229 (58.3%) | 170 (43.3%) | 190 (48.3%) | 156 (39.7%) |
| Sex, male (n = 262) | 152 (58.0%) | 181 (69.1%) | 125 (47.7%) | 136 (51.9%) | 155 (59.2%) | 115 (43.9%) | 128 (48.9%) | 107 (40.8%) |
| Comorbidities |  |  |  |  |  |  |  |  |
| Diabetes mellitus (n = 132) | 82 (62.1%) | 96 (72.7%) | **74 (56.1%)** | 74 (56.1%) | 81 (61.4%) | 51 (38.6%) | 57 (43.2%) | 52 (39.4%) |
| Malignancy (n = 97) | **65 (67.0%)** | **79 (81.4%)** | **55 (56.7%)** | **60 (61.9%)** | **67 (69.1%)** | 35 (36.1%) | **38 (39.2%)** | 31 (32.0%) |
| Liver disease^a^ (n = 81) | **59 (72.8%)** | **68 (84.0%)** | 42 (51.9%) | **50 (61.7%)** | **57 (70.4%)** | 31 (38.3%) | **30 (37.0%)** | 22 (27.2%) |
| End-stage renal disease (n = 73) | 40 (54.8%) | 56 (76.7%) | 37 (50.7%) | 41 (56.2%) | 46 (63.0%) | 38 (52.1%) | 39 (53.4%) | 36 (49.3%) |
| Chronic lung disease^b^ (n = 61) | 39 (63.9%) | 45 (73.8%) | 28 (45.9%) | 33 (54.1%) | 39 (63.9%) | 21 (34.4%) | 24 (39.3%) | 19 (31.1%) |
| Congestive heart failure (n = 50) | 23 (46.0%) | 33 (66.0%) | 22 (44.0%) | 24 (48.0%) | 29 (58.0%) | 25 (50.0%) | 24 (48.0%) | 20 (40.0%) |
| Immunocompromised^c^ (n = 53) | 26 (49.1%) | 35 (66.0%) | 21 (39.6%) | 26 (49.1%) | 26 (49.1%) | 17 (32.1%) | 24 (45.3%) | 18 (34.0%) |
| Total parenteral nutrition (n = 46) | 30 (65.2%) | 34 (73.9%) | **30 (65.2%)** | 30 (65.2%) | 32 (69.6%) | 18 (39.1%) | 19 (41.3%) | 17 (37.0%) |
| Antibiotic exposure (n = 184) | 101 (54.9%) | 118 (64.1%) | 93 (50.5%) | 98 (53.3%) | 110 (59.8%) | 70 (38.0%) | **75 (40.8%)** | **55 (29.9%)** |
| Infection focus |  |  |  |  |  |  |  |  |
| Pneumonia (n = 167) | 100 (59.9%) | 117 (70.1%) | 84 (50.3%) | 92 (55.1%) | **107 (64.1%)** | 64 (38.3%) | **68 (40.7%)** | **54 (32.3%)** |
| Catheter-related infection (n = 152) | 84 (55.3%) | 101 (66.4%) | 68 (44.7%) | 75 (49.3%) | **79 (52.0%)** | 71 (46.7%) | **84 (55.3%)** | **72 (47.4%)** |
| Intraabdominal infection (n = 34) | 23 (67.6%) | 26 (76.5%) | 19 (55.9%) | 21 (61.8%) | 24 (70.6%) | 17 (50.0%) | 14 (41.2%) | 13 (38.2%) |
| Urinary tract infection (n = 12) | 4 (33.3%) | 6 (50.0%) | 4 (33.3%) | 4 (33.3%) | 5 (41.7%) | 6 (50.0%) | 7 (58.3%) | 5 (41.7%) |
| Soft tissue infection (n = 13) | 5 (38.5%) | 8 (61.5%) | 8 (61.5%) | 5 (38.5%) | 7 (53.8%) | 6 (46.2%) | 9 (69.2%) | 6 (46.2%) |
| Shock (n = 174) | **110 (63.2%)** | **127 (73.0%)** | **97 (55.7%)** | **105 (60.3%)** | **117 (67.2%)** | **61 (35.1%)** | **68 (39.1%)** | **46 (26.4%)** |
| Invasive mechanical ventilation (n = 371) | 208 (56.1%) | 251 (67.7%) | 177 (47.7%) | 191 (51.5%) | 215 (58.0%) | 161 (43.4%) | 181 (48.8%) | 150 (40.4%) |
| Antibiotic regimen^d^ |  |  |  |  |  |  |  |  |
| Sulbactam (n = 51) | **19 (37.3%)** | **27 (52.9%)** | 18 (35.3%) | **17 (33.3%)** | **21 (41.2%)** | 28 (54.9%) | **32 (62.7%)** | 26 (51.0%) |
| Colistin (n = 163) | 88 (54.0%) | 116 (71.2%) | **57 (35.0%)** | **73 (44.8%)** | 86 (52.8%) | **83 (50.9%)** | **95 (58.3%)** | 73 (44.8%) |
| Aminoglycoside (n = 21) | 13 (61.9%) | 16 (76.2%) | 8 (38.1%) | 10 (47.6%) | 13 (61.9%) | **4 (19.0%)** | 10 (47.6%) | 9 (42.9%) |
| Carbapenam (n = 169) | 99 (58.6%) | **125 (74.0%)** | 74 (43.8%) | 86 (50.9%) | 99 (58.6%) | 72 (42.6%) | 89 (52.7%) | 71 (42.0%) |
| Tigecycline (n = 80) | **37 (46.3%)** | 51 (63.7%) | 34 (42.5%) | 38 (47.5%) | 43 (53.8%) | 36 (45.0%) | 40 (50.0%) | 34 (42.5%) |
| ^a^ Chronic hepatitis or liver cirrhosis.  ^b^ Asthma, chronic obstructive pulmonary disease, interstitial lung disease, bronchiectasis, or active tuberculosis.  ^c^ Chronic steroid use (prednisolone 5 mg/day or equivalent > 1 month or > 30 mg/day) or other immunosuppressive therapy for diseases such as connective tissue disease, rheumatic disease, or solid organ transplantation.  ^d^ Only antibiotics used for more than 2 days and initiated within 3 days of the bloodstream infection onset date were included.  **Boldface** indicates a *p* value< 0.05 from the chi-square test. | | | | | | | | |

**Supplementary figure legends**

**Fig. S1.** Study algorithm. BSI, bloodstream infection; CRAB, Carbapenem-resistant *Acinetobacter baumannii*; CRGNB, Carbapenem-resistant gram-negative bacteria


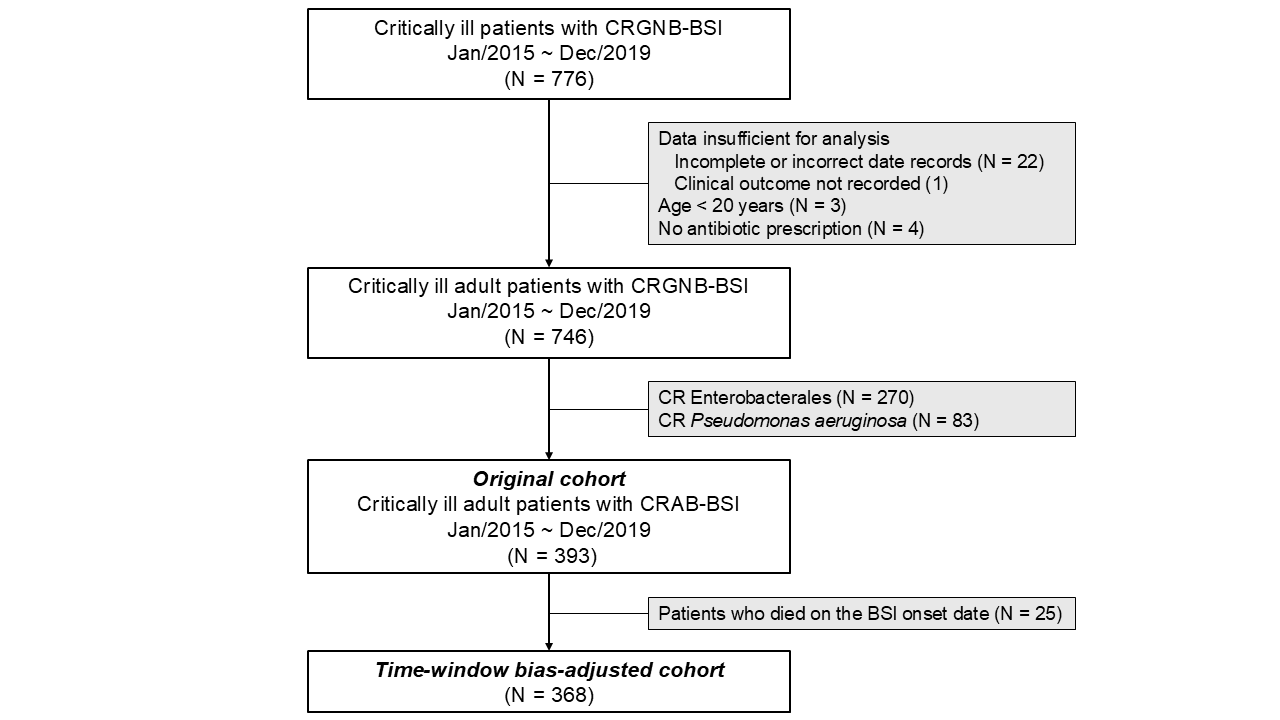


STROBE Statement—Checklist of items that should be included in reports of ***cohort studies***

|  | Item No | Recommendation | Page No |
| --- | --- | --- | --- |
| **Title and abstract** | 1 | (*a*) Indicate the study’s design with a commonly used term in the title or the abstract | 1,4 |
|  |  | (*b*) Provide in the abstract an informative and balanced summary of what was done and what was found | 4,5 |
| Introduction | | | |
| Background/rationale | 2 | Explain the scientific background and rationale for the investigation being reported | 6,7 |
| Objectives | 3 | State specific objectives, including any prespecified hypotheses | 7 |
| Methods | | | |
| Study design | 4 | Present key elements of study design early in the paper | 8 |
| Setting | 5 | Describe the setting, locations, and relevant dates, including periods of recruitment, exposure, follow-up, and data collection | 8 |
| Participants | 6 | (*a*) Give the eligibility criteria, and the sources and methods of selection of participants. Describe methods of follow-up | 8 |
|  |  | (*b*) For matched studies, give matching criteria and number of exposed and unexposed | NA |
| Variables | 7 | Clearly define all outcomes, exposures, predictors, potential confounders, and effect modifiers. Give diagnostic criteria, if applicable | 8-11 |
| Data sources/ measurement | 8* | For each variable of interest, give sources of data and details of methods of assessment (measurement). Describe comparability of assessment methods if there is more than one group | 10-11 |
| Bias | 9 | Describe any efforts to address potential sources of bias | 10-12 |
| Study size | 10 | Explain how the study size was arrived at | 10 |
| Quantitative variables | 11 | Explain how quantitative variables were handled in the analyses. If applicable, describe which groupings were chosen and why | 11-12 |
| Statistical methods | 12 | (*a*) Describe all statistical methods, including those used to control for confounding | 11-12 |
|  |  | (*b*) Describe any methods used to examine subgroups and interactions | 11-12 |
|  |  | (*c*) Explain how missing data were addressed | 12 |
|  |  | (*d*) If applicable, explain how loss to follow-up was addressed | NA |
|  |  | (*e*) Describe any sensitivity analyses | NA |
| Results | | |  |
| Participants | 13* | (a) Report numbers of individuals at each stage of study—eg numbers potentially eligible, examined for eligibility, confirmed eligible, included in the study, completing follow-up, and analysed | 13 |
|  |  | (b) Give reasons for non-participation at each stage | Fig. S1 |
|  |  | (c) Consider use of a flow diagram | Fig. S1 |
| Descriptive data | 14* | (a) Give characteristics of study participants (eg demographic, clinical, social) and information on exposures and potential confounders | 13 |
|  |  | (b) Indicate number of participants with missing data for each variable of interest | Table 1 |
|  |  | (c) Summarise follow-up time (eg, average and total amount) | NA |
| Outcome data | 15* | Report numbers of outcome events or summary measures over time | Table S5 |
| Main results | 16 | (*a*) Give unadjusted estimates and, if applicable, confounder-adjusted estimates and their precision (eg, 95% confidence interval). Make clear which confounders were adjusted for and why they were included | 14-15  Table 2 |
|  |  | (*b*) Report category boundaries when continuous variables were categorized | 12  Fig. 2 |
|  |  | (*c*) If relevant, consider translating estimates of relative risk into absolute risk for a meaningful time period | NA |
| Other analyses | 17 | Report other analyses done—eg analyses of subgroups and interactions, and sensitivity analyses | 15 |
| Discussion | | | |
| Key results | 18 | Summarise key results with reference to study objectives | 17-21 |
| Limitations | 19 | Discuss limitations of the study, taking into account sources of potential bias or imprecision. Discuss both direction and magnitude of any potential bias | 20-21 |
| Interpretation | 20 | Give a cautious overall interpretation of results considering objectives, limitations, multiplicity of analyses, results from similar studies, and other relevant evidence | 17-21 |
| Generalisability | 21 | Discuss the generalisability (external validity) of the study results | 21 |
| Other information | | | |
| Funding | 22 | Give the source of funding and the role of the funders for the present study and, if applicable, for the original study on which the present article is based | 24 |

*Give information separately for exposed and unexposed groups.

**Note:** An Explanation and Elaboration article discusses each checklist item and gives methodological background and published examples of transparent reporting. The STROBE checklist is best used in conjunction with this article (freely available on the Web sites of PLoS Medicine at http://www.plosmedicine.org/, Annals of Internal Medicine at http://www.annals.org/, and Epidemiology at http://www.epidem.com/). Information on the STROBE Initiative is available at http://www.strobe-statement.org.
